# Identification of C3 as a therapeutic target for diabetic nephropathy by bioinformatics analysis

**DOI:** 10.1038/s41598-020-70540-x

**Published:** 2020-08-10

**Authors:** ShuMei Tang, XiuFen Wang, TianCi Deng, HuiPeng Ge, XiangCheng Xiao

**Affiliations:** Department of Nephrology, XiangYa Hospital, Central South University, XiangYa Road NO 87, Changsha, 41008 Hunan China

**Keywords:** Computational biology and bioinformatics, Data integration, Data mining, Predictive medicine

## Abstract

The pathogenesis of diabetic nephropathy is not completely understood, and the effects of existing treatments are not satisfactory. Various public platforms already contain extensive data for deeper bioinformatics analysis. From the GSE30529 dataset based on diabetic nephropathy tubular samples, we identified 345 genes through differential expression analysis and weighted gene coexpression correlation network analysis. GO annotations mainly included neutrophil activation, regulation of immune effector process, positive regulation of cytokine production and neutrophil-mediated immunity. KEGG pathways mostly included phagosome, complement and coagulation cascades, cell adhesion molecules and the AGE-RAGE signalling pathway in diabetic complications. Additional datasets were analysed to understand the mechanisms of differential gene expression from an epigenetic perspective. Differentially expressed miRNAs were obtained to construct a miRNA-mRNA network from the miRNA profiles in the GSE57674 dataset. The miR-1237-3p/SH2B3, miR-1238-5p/ZNF652 and miR-766-3p/TGFBI axes may be involved in diabetic nephropathy. The methylation levels of the 345 genes were also tested based on the gene methylation profiles of the GSE121820 dataset. The top 20 hub genes in the PPI network were discerned using the CytoHubba tool. Correlation analysis with GFR showed that *SYK*, *CXCL1, LYN, VWF*, *ANXA1*, *C3*, *HLA-E, RHOA*, *SERPING1*, *EGF* and *KNG1* may be involved in diabetic nephropathy. Eight small molecule compounds were identified as potential therapeutic drugs using Connectivity Map.

## Introduction

It is estimated that a total of 451 million people suffered from diabetes by 2017, and the number is speculated to be 693 million by 2045^1^. As one of the most serious microvascular complications, diabetic nephropathy (DN) has been a major cause of end-stage renal disease (ESRD) in many countries. The congregation of advanced glycation end-products, oxidative stress and activation of protein kinase C are the major pathogeneses of DN. A new viewpoint holds that tubular injury plays an important and even initial role^2^. Current treatment strategies for DN aim at controlling blood glucose and blood pressure levels and inhibiting the RAS system to reduce albuminuria and delay the progression of DN^3^. However, considering the high incidence of DN-related ESRD, the effect is not entirely satisfactory. Therefore, there is a critical need to identify new therapeutic targets and improve clinical management.

High-throughput sequencing technology offers an effective method to study disease-related genes and provides promising medication goals in many fields^4^. To date, several studies have screened genes or miRNAs involved in DN^5–9^. Integrating these data could overcome the heterogeneity of studies and provide more accurate information. This study identified target genes that may improve the understanding of the molecular mechanisms of DN and provide a resource to build new hypotheses for further follow-up studies. We suggest that the complement system may serve as a therapeutic target in DN.

## Results

### Differential expression analysis of genes in the GSE30529 dataset

**Figure 1:**
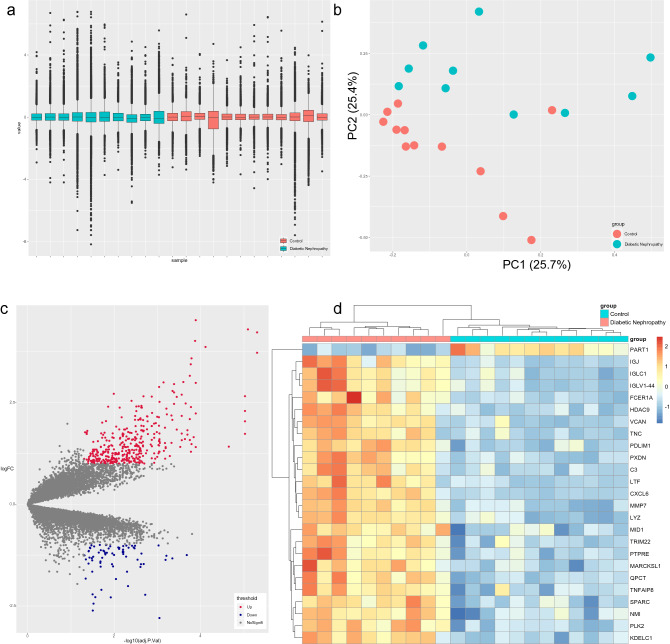
Differential expression analysis of GSE30529. (**a**) Boxplot of GSE30529. (**b**) PCA of GSE30529. The two main components contributed 25.7% and 25.4%. (**c**) Volcano map of DEGs. A total of 386 upregulated DEGs and 71 downregulated DEGs were identified between the DN group and the control group with the criteria of |log2 FC| greater than 1 and adjusted *P* value less than 0.05. (**d**) Heatmap of the top 25 DEGs.

Differential expression analysis of genes in the GSE30529 dataset^5^ was performed to obtain differentially expressed genes (DEGs) that may be involved in DN. First, the GSE30529 dataset was subjected to quality examination to detect batch effects and determine the principal component of the dataset that contributed the most to the variance. The boxplot showed that the overall gene expression levels of the samples in the GSE30529 dataset were approximately the same (Fig. 1a), suggesting that there was no batch effect. In addition, the two main components contributed 25.7% and 25.4% in principal component analysis (PCA) (Fig. 1b), suggesting that there are obviously different components between the DN group and the control group. These different components may be biologically significant DEGs. After the quality inspection, differential expression analysis was performed by the limma package^10^ to acquire DEGs with the criteria of |log2-fold change (FC)| greater than 1 and adjusted *p* value less than 0.05. As a result, 386 upregulated DEGs and 71 downregulated DEGs were identified between the DN group and the control group. The volcano map in Fig. 1c displays the general distribution of these genes, and the top 25 DEGs (*PART1*, *IGJ*, *IGLC1*, *IGLV1-44*, *FCER1A*, *HDAC9*, *VCAN*, *TNC*, *PDLIM1*, *PXDN*, *C3*, *LTF*, *CXCL6*, *MMP7*, *LYZ*, *MID1*, *TRIM22*, *PTPRE*, *MARCKSL1*, *QPCT*, *TNFAIP8*, *SPARC*, *NMI*, *PLK2* and *KDELC1*) are shown in the hierarchical clustering heatmap in Fig. 1d.

### Weighted gene coexpression network analysis of the GSE30529 dataset

**Figure 2:**
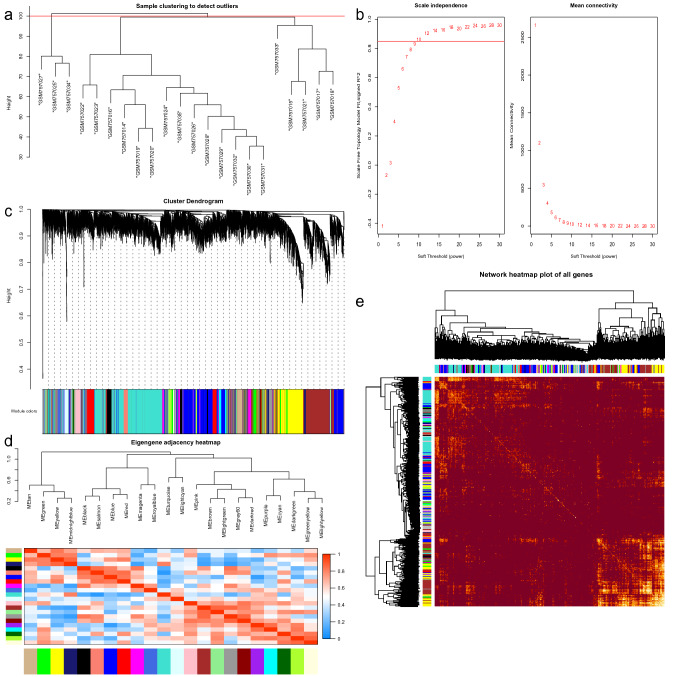
WGCNA of GSE30529. (**a**) Sample clustering of GSE30529. (**b**) Analysis of soft-thresholding powers to fit the scale-free topology model and the mean connectivity of the soft-thresholding powers; 10 was chosen as the value to construct a scale-free network. (**c**) Dendrogram of the gene modules. The branches represent different gene modules, and each leaf represents a gene in the cluster dendrogram. (**d**) Clustering and heatmap of 22 gene modules. (**e**) Heatmap of the weighted gene coexpression correlations of all genes.

**Figure 3:**
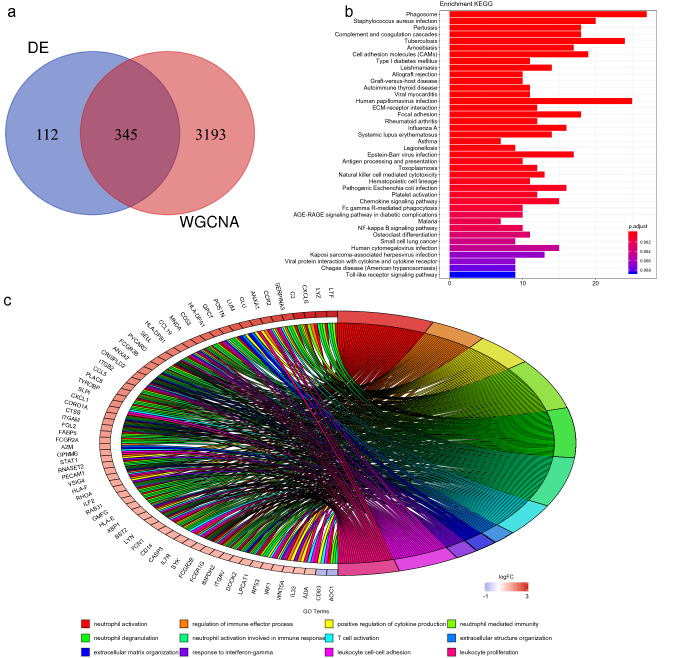
Enrichment analysis. (**a**) Venn diagram of the DEG list and highly related gene list. A total of 345 target genes were obtained. (**b**, **c**) GO annotation and KEGG pathway enrichment analysis. GO annotations mainly included neutrophil activation, regulation of immune effector process, positive regulation of cytokine production and neutrophil-mediated immunity. KEGG pathways mostly included phagosome, complement and coagulation cascades, cell adhesion molecules and ECM-receptor interaction and focal adhesion.

Compared with differential analysis that focuses on the differential expression of genes, the advantage of weighted gene coexpression network analysis (WGCNA) is that it uses expression correlation information between multiple genes to identify genes of interest. Therefore, we applied two analytical methods to screen the target genes. Similarly, we performed sample cluster analysis first to learn sample similarity. The results showed that there were 3 outliers (Fig. 2a); therefore, three samples (GSM757025, GSM757027 and GSM757034) were removed. When performing WGCNA, to construct a scale-free network, the scale-free topological fitting index reaches 0.85 and the mean connectivity reaches 100 by setting the soft threshold power value to 10 (Fig. 2b). Based on the weighted gene coexpression correlation, hierarchical clustering analysis was carried out to obtain different gene modules, which are represented by branches of the clustering tree and different colours. A total of 22 modules were found in the network, with module sizes ranging from 30 to 10,000 and merge cut hights of 0.25 (Fig. 2c). The 22 modules were divided into two clusters in general according to the relationships between the modules (Fig. 2d). In addition, the weighted coexpression correlations of all genes were displayed in a heatmap plot (Fig. 2e). Finally, 3,538 highly related genes were selected in the TOM matrix with a threshold greater than 0.1. The results of the two analyses can be combined to obtain more accurate targets. Therefore, a list of 345 target genes was obtained, and these genes may play a regulatory role in DN (Fig. 3a).

### Functional enrichment analysis of the target genes

The pathogenesis of diabetic nephropathy is very complex, and understanding the functions of the target genes could guide the direction of new research. Functional enrichment analysis of the target genes was performed with the clusterProfiler package^11^ to explore the Gene Ontology (GO) annotations and Kyoto Encyclopedia of Genes and Genomes (KEGG) pathways in which target genes are involved. The top 12 GO terms were identified and mainly included neutrophil activation, regulation of immune effector process, positive regulation of cytokine production and neutrophil-mediated immunity (Fig. 3b). The KEGG pathways mostly included phagosome, complement and coagulation cascades, cell adhesion molecules (CAMs), ECM-receptor interaction and focal adhesion (Fig. 3c). The AGE-RAGE signalling pathway in diabetic complications was also found. It is interesting that the immune system seems to play an important role.

### Potential epigenetic regulatory mechanism

It has now been recognized that the occurrence and development of DN are the result of complex interactions between genetic and environmental factors. Environmental signals could change intracellular pathways through chromatin modifiers and regulate gene expression patterns leading to diabetes and its complications^12^. After determining the target genes, we studied more datasets to understand the potential mechanisms of the differential expression of the target genes, including the GSE51674 dataset^9^, which contains miRNA profiles, and the GSE121820 dataset, which contains DNA methylation profiles.

**Figure 4:**
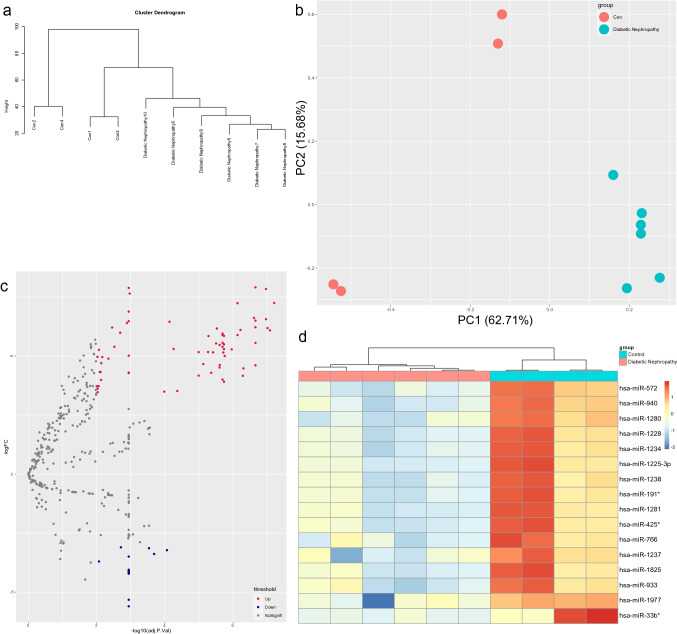
Differential expression analysis of GSE51674. (**a**) Cluster dendrogram of GSE51674. (**b**) Principal component analysis of GSE30529. The two main components contributed 62.71% and 15.68%. (**c**) Volcano map of differentially expressed miRNAs. Sixty-seven upregulated miRNAs and 16 downregulated miRNAs were identified between the DN group and the control group with the criteria of |log2 FC| greater than 3 and adjusted *p* value less than 0.01. (**d**) Heatmap of the downregulated DEGs.

**Figure 5:**
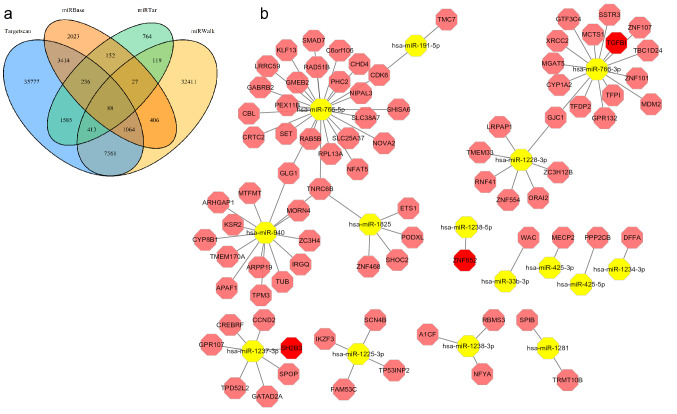
miRNA-mRNA network. (**a**) Venn plot of four prediction results. (**b**) miRNA-mRNA network. In this network, *TGFBI*, *SH2B3* and *ZNF652* in red were upregulated in GSE30529.

Generally, gene expression could be inhibited by miRNAs via base pairing with mRNA. Differential expression analysis was performed on the miRNA profiles of the GSE51674 dataset^9^. Similarly, quality examinations of GSE51674 were performed. There were no very heterogeneous samples in the sample cluster dendrogram (Fig. 4a). PCA showed that the two main components contributed 62.71% and 15.68%, respectively (Fig. 4b). Next, 16 downregulated miRNAs and 67 upregulated miRNAs were found with the criteria of |log2 FC| greater than 3 and adjusted *p* value less than 0.01 (Fig. 4c). The 16 downregulated miRNAs are shown in the hierarchical clustering heatmap in Fig. 4d. To construct a downregulated miRNA-mRNA network, the TargetScan, miRWalk, miRBase and miRTarBase databases^13–16^ were used for target gene prediction of the miRNAs. Eighty-eight downregulated miRNA-mRNA pairs were obtained according to the miRNA target webtools (Fig. 5a). Among them, *TGFBI*, *SH2B3* and *ZNF652* were upregulated in the GSE30529 dataset (Fig. 5b). Therefore, the miR-1237-3p/SH2B3, miR-1238-5p/ZNF652 and miR-766-3p/TGFBI axes may be involved in diabetic nephropathy. Similar work was carried out on the upregulated miRNAs, but their predicted genes did not overlap with the target genes from GSE30529.

DNA methylation is the main epigenetic form of gene expression regulation. To understand the methylation level changes of the target genes, the GSE121820 dataset was downloaded as a validation dataset. Among 345 target genes, 227 genes had methylation differences between the DN group and the control group (Supplemental Table 1).

**Table 1:** Small molecular compounds identified by connectivity map.

Cell ID molecule	HEPG2	HA1E	A375	HT29	PC3	HCC515	A549	VCAP	MCF7	Summary
VEGF-receptor-2-kinase-inhibitor-IV	NaN	− 97.65	NaN	NaN	− 98.72	− 36.65	NaN	39.31	NaN	− 95.76
GPR158	− 99.67	− 99.54	− 87.54	− 85.11	− 95.37	− 96.24	NaN	0.00	− 78.44	− 99.00
CYP51A1	− 96.01	− 25.96	− 98.71	66.44	− 91.52	NaN	− 99.55	NaN	0.00	− 99.38
PTMS	NaN	0.00	− 98.98	− 93.33	− 78.99	− 96.77	− 98.10	− 35.22	0.00	− 97.04
Withaferin-A	0.00	0.00	− 92.00	− 14.15	− 97.00	− 98.40	0.00	− 90.66	− 96.15	− 95.84
Digoxin	NaN	0.00	− 98.81	0.00	− 76.95	− 97.80	0.00	− 91.20	− 97.67	− 96.35
Digoxin	0.00	0.00	− 97.41	0.00	− 96.72	− 83.92	− 93.40	− 98.91	− 87.77	− 95.77
Ouabain	− 91.46	− 25.32	− 96.72	− 88.99	− 89.54	− 53.35	− 97.20	0.00	− 92.89	− 94.13
PHF15	64.27	− 81.91	− 95.04	0.00	− 96.29	NaN	− 96.54	NaN	0.00	− 95.50

### PPI network and identification of hub genes

**Figure 6:**
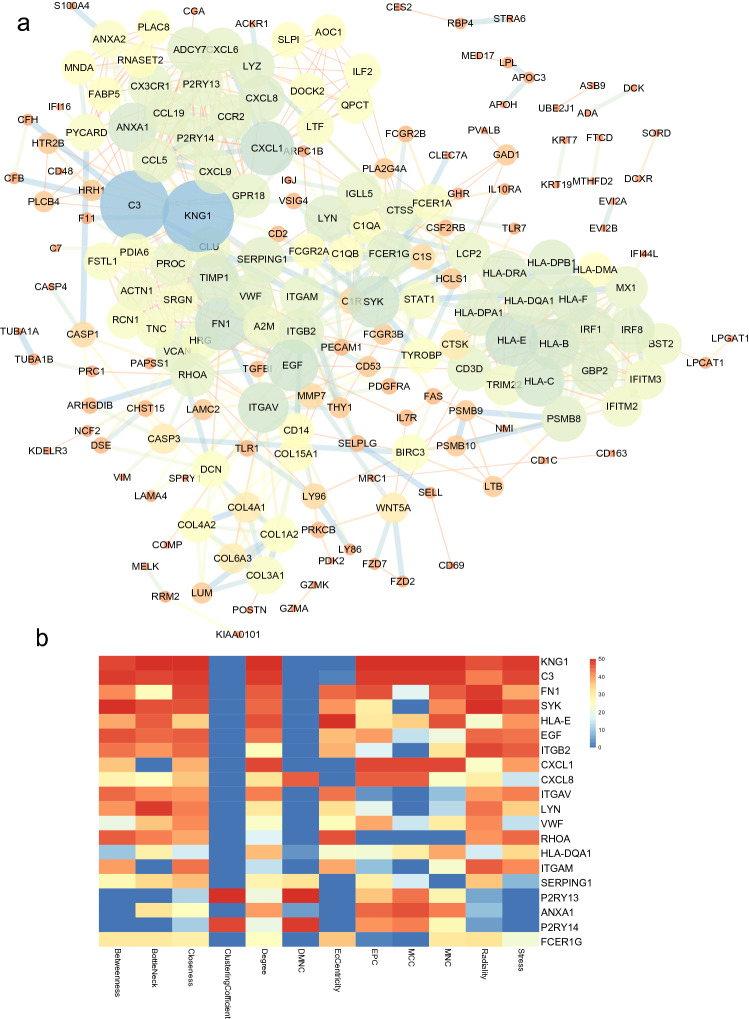
PPI network. (**a**) PPI network of combined genes. There are 190 nodes and 680 edges. The size and gradient colour of nodes are adjusted by degree. The thickness and gradient colour of the edge are adjusted by the interaction score. (**b**) Heatmap of the CytoHubba analysis score.

First, the list of target genes was exported to the STRING database. By setting the interaction confidence score at the highest level at 0.9, a protein–protein interaction (PPI) network was constructed, which contained 190 nodes and 680 edges (Fig. 6a). Each node represents a protein, and an edge represents an interaction between proteins. The size and gradient colour of the nodes are adjusted by the degree, while the thickness and gradient colour of the edge are adjusted by the interaction score. To search for important nodes in the networks, all nodes were ranked by the 12 topological analysis methods provided by CytoHubba. Each algorithm computed all node scores, and then 1–50 points were assigned based on the rank. According to all points, the top 20 nodes (KNG1, C3, FN1, SYK, HLA-E, EGF, ITGB2, CXCL1, CXCL8, ITGAV, LYN, VWF, RHOA, HLA-DQA1, ITGAM, SERPING1, P2RY13, ANXA1, P2RY14 and FCER1G) were identified (Fig. 6b). Because the products of genes were at the core of the PPI network, these hub genes were considered potential therapeutic targets.

### Clinical data validation and drug prediction

**Figure 7:**
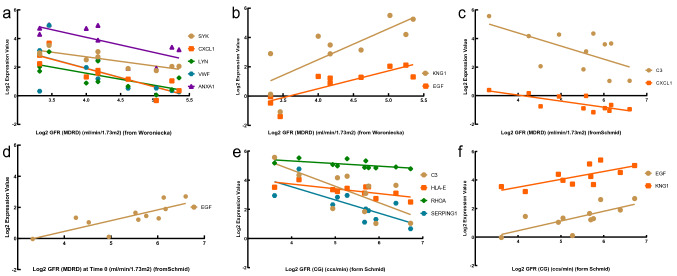
Pearson correlation analyses of GFR and target genes. (**a**) The gene expression of *SYK* (*p* = 0.0022, r =  − 0.8437), *CXCL1* (*p* = 0.0016, r =  − 0.8554), *LYN* (*p* = 0.0269, r =  − 0.6911), *VWF* (*p* = 0.0452, r =  − 0.6423) and *ANXA1* (*p* = 0.0211, r =  − 0.7111) was negatively related to GFR. (**b**) The gene expression of EGF (*p* = 0.0027, r = 0.8349) and KNG1 (*p* = 0.0073, r = 0.7838) was positively correlated with GFR. (**c**) The gene expression of *C3* (*p* = 0.0459, r =  − 0.6109) and *CXCL1* (*p* = 0.0061, r =  − 0.7645) was negatively correlated with GFR. (**d**) The gene expression of EGF (*p* = 0.0037, r = 0.7919) was positively related to GFR. (**e**) The gene expression of *C3* (*p* = 0.0171, r =  − 0.6970), *HLA-E* (*p* = 0.0132, r =  − 0.7161), *RHOA* (*p* = 0.0439, r =  − 0.6154) and *SERPING1* (*p* = 0.0091, r =  − 0.7409) was negatively correlated with GFR. (**f**) *EGF* (*p* = 0.0121, r = 0.7221) and *KNG1* (*p* = 0.0153, r = 0.7053) were positively related to GFR.

To verify the potential roles of the hub genes in DN, clinical data including two datasets (Woroniecka and Schmid) from Nephroseq were obtained, and Pearson correlation analysis was performed between the hub genes and clinical data. The gene expression of *SYK*, *CXCL1, LYN, VWF*, *ANXA1*, *C3*, *HLA-E, RHOA* and *SERPING1* in DN tubule samples was negatively related to GFR, suggesting a pathogenic role of the upregulated genes (Fig. 7a, c, e). Conversely, the gene expression of *EGF* and *KNG1* in DN tubule samples was positively related to GFR, suggesting a protective role of the downregulated genes (Fig. 7b, d, f).

Given that the effectiveness of existing treatment strategies is not entirely satisfactory, it is necessary to propose new strategies and develop new therapeutic methods. Connectivity Map^17^ was used to compare the DEG list with the database reference dataset, and a correlation score (− 100 to 100) was obtained. Negative numbers indicate that the DEG list and the reference gene expression spectrum may be opposite; that is, the expression spectrum of drug disturbance is negatively correlated with the expression spectrum of disease disturbance. Twenty-three upregulated DEGs (logFC greater than 2.5) and 13 downregulated DEGs (logFC less than 1.5) were exported to Connectivity Map to search for potential drugs. Small molecule compounds with an average coefficient of less than − 90 were sorted according to the correlation score of the reference gene expression spectrum. As a result, 8 small molecule compounds were identified as potential therapeutic drugs (Table 1).

## Discussion

As one of the microvascular complications of diabetes, DN is the main cause of ESRD. Existing treatments are not sufficient to control the development of disease. New treatment strategies are needed. High-throughput omics data have been widely used to study the mechanisms of disease and predict possible therapeutic targets. We performed differential expression analysis and WGCNA of GSE30529 and obtained 345 target genes. GO annotations mainly included neutrophil activation, regulation of immune effector process, positive regulation of cytokine production and neutrophil-mediated immunity. KEGG pathways mostly included phagosome, complement and coagulation cascades, cell adhesion molecules (CAMs), ECM-receptor interaction, focal adhesion and AGE-RAGE signalling pathway in diabetic complications. The results supported that the immune response may be involved in DN. Cytokine release and extracellular matrix deposition may be subsequent events and continue with the development of disease. We also studied additional datasets to understand the potential mechanisms of the differential expression of the target genes. The miRNA-mRNA network suggested that the miR-766-3p/TGFBI, miR-1238-5p/ZNF652 and miR-1237-3p/SH2B3 axes may be involved in diabetic nephropathy and that most target genes have differences in DNA methylation levels between the DN group and the control group. Next, a PPI network was established, and the 20 hub genes were identified. Furthermore, correlation analysis with clinical data demonstrated the disease-promoting effect of *SYK*, *CXCL1, LYN, VWF*, *ANXA1*, *C3*, *HLA-E, RHOA* and *SERPING1*, which were upregulated in DN tubule samples. In contrast, *EGF* and *KNG1*, which were downregulated in DN tubule samples, were suggested to have protective effects in DN.

To date, there have been some reports about hub genes and DN. Spleen tyrosine kinase (SYK) was reported to mediate high glucose-induced TGF-β1 and IL-1β secretion^18,19^. In a diabetic animal model, C-X-C motif chemokine ligand 1 (CXCL1) was found to possibly serve as a proinflammatory mediator^20,21^. In addition, VWF was reported to be involved in intrarenal thrombosis leading to the deterioration of renal function^22^. Purvis et al. observed higher circulating plasma levels of ANXA1 in T1D and T2D patients, whereas the exogenous supplementation of ANXA1 improves insulin resistance and prevents the progression of subsequent microvascular complications in mice^23,24^. Previous studies have demonstrated that statins prevent DN by reducing the activity of Ras homolog family member A (RhoA) protein activation^25–28^. Another study reported that the activation of RhoA/ROCK may regulate the NF-κB signalling pathway^29^. In addition, sinomenine, kaempferol, catalpol and rutin have been shown to have protective effects through the RhoA/ROCK signalling pathway^30–33^. EGF was considered a urine biomarker in two studies^34,35^. Recently, the newest report about cytosine methylation differences in kidney tubule samples supported this viewpoint^36^. In addition, one large-scale linkage study revealed polymorphisms in kininogen 1 (KNG1) associated with DN in European populations^37^.

C3 was the gene of interest through differential expression analysis and WGCNA. The KEGG pathways of the target genes also included the complement and coagulation cascade. In addition, the selection of the core genes in the PPI network also indicated that C3 was centrally located. These results may prove that complement C3 serves as a therapeutic target in diabetic nephropathy. The results are consistent with knowledge that the complement system participates in DN. The development of diabetes is intimately linked to low-grade inflammation^38^. High levels of inflammatory markers such as C-reactive protein and adiponectin proved this viewpoint^39,40^. Inflammation might promote the occurrence and development of diabetic complications such as DN. However, the underlying mechanisms of the initiation of low-grade inflammation are still poorly understood. Increasing research evidence has proven that the innate immune system is closely involved in diabetes^41^. Simultaneously, the roles for pattern recognition receptors (PRRs) associated with DN have been discussed^42,43^. The complement system is not only involved in innate immune defence by PRRs (mannose-binding lectin and ficolin) but also considered an important proinflammatory factor. Several studies have pointed out that the complement system is involved in the pathogenesis of DN and might be a therapeutic target^44–46^. Significant differences in complement system component levels in both plasma and urine were found between DN patients and diabetic patients. In addition, Li et al. highlighted the relatively more important impact of C3a, C5a and sC5b-9 in the development of DN^47^. Sun et al. demonstrated that more severe kidney damage was associated with the deposition of C1q and C3c in renal histopathology assessment^48^. Furthermore, a large-scale cohort study substantiated that diabetic patients with high plasma levels of C3 are more prone to kidney damage than the general population^49^. Another study indicated that the serum levels of C3 may help to differentiate DN patients from diabetic patients without kidney damage^50^. Blockade of C3a and C5a receptors in a T1DM model indicated a potential protective effect on renal fibrosis by improving endothelial-to-myofibroblast transition through the Wnt/β-catenin signalling pathway^51^. Similarly, blockade of C3a receptors in rats with T2DM improved renal morphology and function by inhibiting cytokine release and TGFβ/Smad3 signalling^52^. However, the best approach for targeting the complement system to prevent the development of DN still needs to be explored. Therefore, 8 potential small molecule compounds were identified by the Connectivity Map database in our study.

In summary, our study has important significance in understanding the underlying mechanisms of DN and is helpful for developing new treatment strategies for DN. However, further molecular biological experiments are needed to verify the association between the identified genes and DN.

## Materials and methods

### Data download

The GSE30529 (expression profiling by array)^5^ and GSE51674 (non-coding RNA profiling by array)^9^ datasets were downloaded by the GEOquery package^53^ in R software version 3.6.2. GSE121820_T2DN-CTL (methylation profiling by genome tiling array, unpublished) was downloaded from the GEO database (https://www.ncbi.nlm.nih.gov/geo/). The GSE30529 dataset based on the GPL571 platform includes 10 DN tubule samples and 12 control samples. The GSE51674 dataset based on the GPL10656 platform includes 6 DN tissue samples and 4 control samples. The GSE121820 dataset based on the GPL5082 platform contains 10 T2 DN blood samples and 10 control samples.

### Data processing

All differential analyses were performed by the limma package^10^. Adjusted *p* values less than 0.05 and |log2-fold change (FC)| greater than 1 were considered statistically significant in the differential analysis of GSE30529. Adjusted *p* values less than 0.01 and |log2 FC| greater than 3 were considered statistically significant in the differential analysis of GSE51674. In addition, the TargetScan, miRWalk, miRBase and miRTarBase databases^13–16^ were used for the target gene prediction of the differentially expressed miRNAs.

Weighted gene coexpression network analysis (WGCNA) allows biologically meaningful module information mining based on pairwise correlations between genes in high-throughput data using the WGCNA package^54^. The WGCNA workflow consists of gene coexpression network construction, module identification, module relationship analysis and the identification of highly related genes. The gene coexpression network was constructed with the filtering principle that the soft threshold makes the network more consistent with a scale-free topology. The modules were identified with the criterion of module size 30–10,000, merge cut height equal to 0.25 and verbose equal to 3. Highly related genes were obtained with thresholds greater than 0.1 in the topological overlap matrix (TOM).

### Functional enrichment analysis and hub gene screening

Gene Ontology (GO) annotation and Kyoto Encyclopedia of Genes and Genomes (KEGG) pathway enrichment analyses were performed with the clusterProfiler package^11^. The STRING database^55^ (version 11.0, https://string-db.org/) was used to search for interactions between the candidate proteins based on laboratory data, other databases, text mining and predictive bioinformatics data. Cytoscape software was used to visualize the protein–protein interaction (PPI) network and perform network analysis. CytoHubba, a built-in tool in Cytoscape, uses 12 methods to explore important nodes in biological networks, such as the Degree method (Deg), Maximum Neighborhood Component (MNC), Density of Maximum Neighborhood Component (DMNC), Maximal Clique Centrality (MCC), Closeness, EcCentricity, Radiality, BottleNeck, Stress, Betweenness, Edge Percolated Component (EPC) and ClusteringCofficient^56^.

### Clinical data analysis and drug analysis

The Nephroseq v5 analysis engine (https://v5.nephroseq.org) provides access to gene expression signatures and clinical features. Pearson correlation analysis was performed between genes and GFR^5,57^. Unpaired Student’s t test was used to compare two groups. *P* values less than 0.05 were considered statistically significant. Nonsignificant results are not displayed.

Connectivity Map17, an online database that relates disease, genes, and drugs based on similar or opposite gene expression signatures, was used for potential drug prediction.

## Supplementary information

Supplementary file1

## Data Availability

The GSE30529, GSE51674 and GSE121820 datasets are available in GEO database (https://www.ncbi.nlm.nih.gov/geo/). The R script data used to support the findings of this study are included within the supplementary information file.

## References

[1] Cho NH (2018). IDF Diabetes Atlas: Global estimates of diabetes prevalence for 2017 and projections for 2045. Diabetes Res. Clin. Pract..

[2] Zeni L, Norden AGW, Cancarini G, Unwin RJ (2017). A more tubulocentric view of diabetic kidney disease. J. Nephrol..

[3] Ruggenenti P, Cravedi P, Remuzzi G (2010). The RAAS in the pathogenesis and treatment of diabetic nephropathy. Nat. Rev. Nephrol..

[4] Petryszak R (2014). Expression Atlas update: a database of gene and transcript expression from microarray- and sequencing-based functional genomics experiments. Nucleic Acids Res..

[5] Woroniecka KI (2011). Transcriptome analysis of human diabetic kidney disease. Diabetes.

[6] Shved N (2017). Transcriptome-based network analysis reveals renal cell type-specific dysregulation of hypoxia-associated transcripts. Sci. Rep..

[7] Ju W (2013). Defining cell-type specificity at the transcriptional level in human disease. Genome Res..

[8] Grayson PC (2018). Metabolic pathways and immunometabolism in rare kidney diseases. Ann. Rheum. Dis..

[9] Conserva F (2019). Urinary miRNA-27b-3p and miRNA-1228-3p correlate with the progression of kidney fibrosis in diabetic nephropathy. Sci. Rep..

[10] Ritchie ME (2015). limma powers differential expression analyses for RNA-sequencing and microarray studies. Nucleic Acids Res..

[11] Yu G, Wang LG, Han Y, He QY (2012). clusterProfiler: an R package for comparing biological themes among gene clusters. OMICS.

[12] Singh S, Sonkar SK, Sonkar GK, Mahdi AA (2019). Diabetic kidney disease: a systematic review on the role of epigenetics as diagnostic and prognostic marker. Diabetes Metab. Res. Rev..

[13] Agarwal V, Bell GW, Nam JW, Bartel DP (2015). Predicting effective microRNA target sites in mammalian mRNAs. Elife.

[14] Sticht C, De La Torre C, Parveen A, Gretz N (2018). miRWalk: an online resource for prediction of microRNA binding sites. PLoS ONE.

[15] Kozomara A, Birgaoanu M, Griffiths-Jones S (2019). miRBase: from microRNA sequences to function. Nucleic Acids Res..

[16] Chou CH (2018). miRTarBase update 2018: a resource for experimentally validated microRNA-target interactions. Nucleic Acids Res..

[17] Subramanian A (2017). A next generation connectivity map: L1000 platform and the first 1,000,000 profiles. Cell.

[18] Yang WS, Chang JW, Han NJ, Lee SK, Park SK (2012). Spleen tyrosine kinase mediates high glucose-induced transforming growth factor-beta1 up-regulation in proximal tubular epithelial cells. Exp. Cell Res..

[19] Qiao Y (2018). Spleen tyrosine kinase promotes NLR family pyrin domain containing 3 inflammasomemediated IL1beta secretion via cJun Nterminal kinase activation and cell apoptosis during diabetic nephropathy. Mol. Med. Rep..

[20] Niu S (2016). Broad infiltration of macrophages leads to a proinflammatory state in streptozotocin-induced hyperglycemic mice. J. Immunol..

[21] Xu J (2016). Diabetes induced changes in podocyte morphology and gene expression evaluated using GFP transgenic podocytes. Int. J. Biol. Sci..

[22] Dhanesha N (2017). ADAMTS13 retards progression of diabetic nephropathy by inhibiting intrarenal thrombosis in mice. Arterioscler. Thromb. Vasc. Biol..

[23] Purvis GSD (2018). Annexin A1 attenuates microvascular complications through restoration of Akt signalling in a murine model of type 1 diabetes. Diabetologia.

[24] Purvis GSD (2019). Identification of AnnexinA1 as an endogenous regulator of RhoA, and its role in the pathophysiology and experimental therapy of type-2 diabetes. Front. Immunol..

[25] Danesh FR (2002). 3-Hydroxy-3-methylglutaryl CoA reductase inhibitors prevent high glucose-induced proliferation of mesangial cells via modulation of Rho GTPase/p21 signaling pathway: Implications for diabetic nephropathy. Proc. Natl. Acad. Sci. U. S. A..

[26] Zeng L (2005). HMG CoA reductase inhibition modulates VEGF-induced endothelial cell hyperpermeability by preventing RhoA activation and myosin regulatory light chain phosphorylation. Faseb J..

[27] Kolavennu V, Zeng L, Peng H, Wang Y, Danesh FR (2008). Targeting of RhoA/ROCK signaling ameliorates progression of diabetic nephropathy independent of glucose control. Diabetes.

[28] Peng F (2008). RhoA/Rho-kinase contribute to the pathogenesis of diabetic renal disease. Diabetes.

[29] Xie X (2013). Activation of RhoA/ROCK regulates NF-kappaB signaling pathway in experimental diabetic nephropathy. Mol. Cell. Endocrinol..

[30] Yin Q, Xia Y, Wang G (2016). Sinomenine alleviates high glucose-induced renal glomerular endothelial hyperpermeability by inhibiting the activation of RhoA/ROCK signaling pathway. Biochem. Biophys. Res. Commun..

[31] Wang X, Zhao X, Feng T, Jin G, Li Z (2016). Rutin prevents high glucose-induced renal glomerular endothelial hyperpermeability by inhibiting the ROS/Rhoa/ROCK signaling pathway. Planta Med..

[32] Sharma D, Gondaliya P, Tiwari V, Kalia K (2019). Kaempferol attenuates diabetic nephropathy by inhibiting RhoA/Rho-kinase mediated inflammatory signalling. Biomed. Pharmacother..

[33] Chen Y (2019). Catalpol ameliorates podocyte injury by stabilizing cytoskeleton and enhancing autophagy in diabetic nephropathy. Front. Pharmacol..

[34] Satirapoj B, Dispan R, Radinahamed P, Kitiyakara C (2018). Urinary epidermal growth factor, monocyte chemoattractant protein-1 or their ratio as predictors for rapid loss of renal function in type 2 diabetic patients with diabetic kidney disease. BMC Nephrol..

[35] Nowak N (2018). Markers of early progressive renal decline in type 2 diabetes suggest different implications for etiological studies and prognostic tests development. Kidney Int..

[36] Gluck C (2019). Kidney cytosine methylation changes improve renal function decline estimation in patients with diabetic kidney disease. Nat. Commun..

[37] Vionnet N (2006). Analysis of 14 candidate genes for diabetic nephropathy on chromosome 3q in European populations: strongest evidence for association with a variant in the promoter region of the adiponectin gene. Diabetes.

[38] Lontchi-Yimagou E, Sobngwi E, Matsha TE, Kengne AP (2013). Diabetes mellitus and inflammation. Curr. Diabetes Rep..

[39] Mazidi M, Toth PP, Banach M (2018). C-reactive protein is associated with prevalence of the metabolic syndrome, hypertension, and diabetes mellitus in US adults. Angiology.

[40] Bruun JM (2003). Regulation of adiponectin by adipose tissue-derived cytokines: in vivo and in vitro investigations in humans. Am. J. Physiol. Endocrinol. Metab..

[41] Wada J, Makino H (2016). Innate immunity in diabetes and diabetic nephropathy. Nat. Rev. Nephrol..

[42] Mudaliar H, Pollock C, Panchapakesan U (2014). Role of Toll-like receptors in diabetic nephropathy. Clin. Sci. (Lond.).

[43] Du P (2013). NOD2 promotes renal injury by exacerbating inflammation and podocyte insulin resistance in diabetic nephropathy. Kidney Int..

[44] Fortpied J, Vertommen D, Van Schaftingen E (2010). Binding of mannose-binding lectin to fructosamines: a potential link between hyperglycaemia and complement activation in diabetes. Diabetes Metab. Res. Rev..

[45] Acosta J (2000). Molecular basis for a link between complement and the vascular complications of diabetes. Proc. Natl. Acad. Sci. U. S. A..

[46] Flyvbjerg A (2017). The role of the complement system in diabetic nephropathy. Nat. Rev. Nephrol..

[47] Li XQ, Chang DY, Chen M, Zhao MH (2019). Complement activation in patients with diabetic nephropathy. Diabetes Metab..

[48] Sun ZJ (2019). Complement deposition on renal histopathology of patients with diabetic nephropathy. Diabetes Metab..

[49] Rasmussen KL, Nordestgaard BG, Nielsen SF (2018). Complement C3 and risk of diabetic microvascular disease: a cohort study of 95202 individuals from the general population. Clin. Chem..

[50] Zhang J (2019). Serum levels of immunoglobulin G and complement 3 differentiate non-diabetic renal disease from diabetic nephropathy in patients with type 2 diabetes mellitus. Acta Diabetol..

[51] Li L (2015). C3a and C5a receptor antagonists ameliorate endothelial-myofibroblast transition via the Wnt/beta-catenin signaling pathway in diabetic kidney disease. Metabolism.

[52] Li L (2014). C3a receptor antagonist ameliorates inflammatory and fibrotic signals in type 2 diabetic nephropathy by suppressing the activation of TGF-beta/smad3 and IKBalpha pathway. PLoS ONE.

[53] Davis S, Meltzer PS (2007). GEOquery: a bridge between the Gene Expression Omnibus (GEO) and BioConductor. Bioinformatics.

[54] Langfelder P, Horvath S (2008). WGCNA: an R package for weighted correlation network analysis. BMC Bioinform..

[55] Szklarczyk D (2019). STRING v11: protein-protein association networks with increased coverage, supporting functional discovery in genome-wide experimental datasets. Nucleic Acids Res..

[56] Chin CH (2014). cytoHubba: identifying hub objects and sub-networks from complex interactome. BMC Syst. Biol..

[57] Schmid H (2006). Modular activation of nuclear factor-kappaB transcriptional programs in human diabetic nephropathy. Diabetes.

