# Supplementary material for: Identification of C3 as a therapeutic target for diabetic nephropathy by bioinformatics analysis
Source: Sci Rep. 2020 Aug 10;10:13468. doi: 10.1038/s41598-020-70540-x (PMC7417539; doi:10.1038/s41598-020-70540-x)
Supplement: Supplementary file 1 — Supplementary file1 [file 41598_2020_70540_MOESM1_ESM.docx]

Supplement Table 1. DNA methylation difference of target genes

| Gene | P | Gene | P | Gene | P | Gene | P |
| --- | --- | --- | --- | --- | --- | --- | --- |
| ABCC1 | 8.79E-06 | RPS3 | 7.91E-05 | MGP | 1.63E-03 | PDGFRA | 7.00E-03 |
| ANXA2P2 | 8.79E-06 | SORD | 7.91E-05 | MYO1D | 1.63E-03 | PDK2 | 7.00E-03 |
| BIRC3 | 8.79E-06 | SRGN | 7.91E-05 | TPBG | 1.63E-03 | PRKCB | 7.00E-03 |
| CLDN8 | 8.79E-06 | XBP1 | 7.91E-05 | DSE | 6.90E-03 | PSMB9 | 7.00E-03 |
| FCER1G | 8.79E-06 | CLEC4A | 5.10E-04 | LY75 | 6.90E-03 | PXMP2 | 7.00E-03 |
| GUSBP11 | 8.79E-06 | COL3A1 | 5.10E-04 | TSPAN13 | 6.90E-03 | RALYL | 7.00E-03 |
| HOPX | 8.79E-06 | ETNPPL | 5.10E-04 | WFDC2 | 6.90E-03 | RCN1 | 7.00E-03 |
| KIAA1551 | 8.79E-06 | FLI1 | 5.10E-04 | ADCY7 | 7.00E-03 | S100A4 | 7.00E-03 |
| MSL1 | 8.79E-06 | GABRP | 5.10E-04 | ANXA1 | 7.00E-03 | SELPLG | 7.00E-03 |
| P2RY13 | 8.79E-06 | KRT7 | 5.10E-04 | APOBEC3G | 7.00E-03 | SLC39A4 | 7.00E-03 |
| RAB31 | 8.79E-06 | MPHOSPH8 | 5.10E-04 | APOC3 | 7.00E-03 | SLPI | 7.00E-03 |
| SPRY1 | 8.79E-06 | MRC1 | 5.10E-04 | ARSF | 7.00E-03 | TGFBI | 7.00E-03 |
| WNT5A | 8.79E-06 | QPCT | 5.10E-04 | BCLAF1 | 7.00E-03 | TLR1 | 7.00E-03 |
| AGR2 | 1.76E-05 | SOX4 | 5.10E-04 | C3 | 7.00E-03 | TNFAIP8 | 7.00E-03 |
| ARHGDIB | 1.76E-05 | STRA6 | 5.10E-04 | CD163 | 7.00E-03 | VIM | 7.00E-03 |
| CCL5 | 1.76E-05 | THBS2 | 5.10E-04 | CD3D | 7.00E-03 | VWF | 7.00E-03 |
| CD53 | 1.76E-05 | TSKU | 5.10E-04 | CDHR5 | 7.00E-03 | ZNF652 | 7.00E-03 |
| CORO1A | 1.76E-05 | UBE2J1 | 5.10E-04 | CHST15 | 7.00E-03 | BST2 | 7.35E-03 |
| FABP5 | 1.76E-05 | CYTIP | 6.85E-04 | CLEC7A | 7.00E-03 | CD14 | 7.35E-03 |
| HCLS1 | 1.76E-05 | FKBP11 | 6.85E-04 | COL15A1 | 7.00E-03 | CFH | 7.35E-03 |
| HLA-C | 1.76E-05 | HYAL1 | 6.85E-04 | CPA3 | 7.00E-03 | COL4A2 | 7.35E-03 |
| HLA-DPA1 | 1.76E-05 | NMI | 6.85E-04 | CRISPLD2 | 7.00E-03 | CYP27B1 | 7.35E-03 |
| HLA-E | 1.76E-05 | PTPRE | 6.85E-04 | CSF2RB | 7.00E-03 | DOCK2 | 7.35E-03 |
| ILF2 | 1.76E-05 | C7 | 1.05E-03 | CST6 | 7.00E-03 | F11 | 7.35E-03 |
| LPGAT1 | 1.76E-05 | CAPN6 | 1.05E-03 | CSTA | 7.00E-03 | FHL2 | 7.35E-03 |
| NCF2 | 1.76E-05 | IRF8 | 1.05E-03 | CTSK | 7.00E-03 | FZD7 | 7.35E-03 |
| P2RY14 | 1.76E-05 | MAP1B | 1.05E-03 | CXCL9 | 7.00E-03 | IL10RA | 7.35E-03 |
| PAPSS1 | 1.76E-05 | POSTN | 1.05E-03 | DHRS9 | 7.00E-03 | MNDA | 7.35E-03 |
| PECAM1 | 1.76E-05 | PRC1 | 1.05E-03 | DIP2C | 7.00E-03 | SAMSN1 | 7.35E-03 |
| RASSF2 | 1.76E-05 | TUBA1A | 1.05E-03 | EFNB2 | 7.00E-03 | SH2B3 | 7.35E-03 |
| SEL1L3 | 1.76E-05 | BHLHE41 | 1.17E-03 | EVI2B | 7.00E-03 | THY1 | 7.35E-03 |
| SERPINA6 | 1.76E-05 | C1QB | 1.17E-03 | FCGR2B | 7.00E-03 | TNC | 7.35E-03 |
| SKAP2 | 1.76E-05 | CASP3 | 1.17E-03 | FCHSD2 | 7.00E-03 | ACKR1 | 7.82E-03 |
| SLC46A3 | 1.76E-05 | CXCL8 | 1.17E-03 | FN1 | 7.00E-03 | AEBP1 | 7.82E-03 |
| TSPAN1 | 1.76E-05 | DEFB1 | 1.17E-03 | GAD1 | 7.00E-03 | AOC1 | 7.82E-03 |
| VOPP1 | 1.76E-05 | FCGR2A | 1.17E-03 | GBP2 | 7.00E-03 | C1S | 7.82E-03 |
| CX3CR1 | 4.39E-05 | MYOF | 1.17E-03 | GHR | 7.00E-03 | CASP1 | 7.82E-03 |
| FILIP1L | 4.39E-05 | NXN | 1.17E-03 | GPR18 | 7.00E-03 | CD69 | 7.82E-03 |
| IMPDH2 | 4.39E-05 | PSMB8 | 1.17E-03 | HLA-DRA | 7.00E-03 | CD83 | 7.82E-03 |
| FUT3 | 7.03E-05 | SCRN1 | 1.17E-03 | IGSF6 | 7.00E-03 | COL4A1 | 7.82E-03 |
| AGXT | 7.91E-05 | ANXA2 | 1.39E-03 | IRF1 | 7.00E-03 | DCK | 7.82E-03 |
| CASP4 | 7.91E-05 | CFB | 1.39E-03 | ITGAV | 7.00E-03 | DCLK1 | 7.82E-03 |
| CES2 | 7.91E-05 | CGA | 1.59E-03 | ITGB2 | 7.00E-03 | EFHD1 | 7.82E-03 |
| CIDEB | 7.91E-05 | DCN | 1.59E-03 | ITM2C | 7.00E-03 | GZMK | 7.82E-03 |
| CRIP1 | 7.91E-05 | C1R | 1.63E-03 | KDELC1 | 7.00E-03 | IFI16 | 7.82E-03 |
| FCER1A | 7.91E-05 | C1RL | 1.63E-03 | KNG1 | 7.00E-03 | LAMA4 | 7.82E-03 |
| FCN1 | 7.91E-05 | CXCL1 | 1.63E-03 | LAMC2 | 7.00E-03 | LUM | 7.82E-03 |
| IGLL3P | 7.91E-05 | EVI2A | 1.63E-03 | LPCAT1 | 7.00E-03 | PDLIM1 | 7.82E-03 |
| MS4A6A | 7.91E-05 | LCP2 | 1.63E-03 | LTF | 7.00E-03 | PROM1 | 7.82E-03 |
| NR1I3 | 7.91E-05 | RHOA | 1.63E-03 | LY86 | 7.00E-03 | RGS4 | 7.82E-03 |
| PON2 | 7.91E-05 | TRIM16 | 1.63E-03 | LYN | 7.00E-03 | SERPINA3 | 7.82E-03 |
| PROZ | 7.91E-05 | ACOT11 | 1.63E-03 | MARCKSL1 | 7.00E-03 | STAT1 | 7.82E-03 |
| PSMB10 | 7.91E-05 | CLU | 1.63E-03 | MED17 | 7.00E-03 | TES | 7.82E-03 |
| PXDN | 7.91E-05 | FZD2 | 1.63E-03 | MMP7 | 7.00E-03 | TGM2 | 7.82E-03 |
| RBM12 | 7.91E-05 | HTR2B | 1.63E-03 | MOXD1 | 7.00E-03 | TYROBP | 7.82E-03 |
| RBP4 | 7.91E-05 | IL33 | 1.63E-03 | MS4A4A | 7.00E-03 | YLPM1 | 7.82E-03 |
| RPAP3 | 7.91E-05 | IL7R | 1.63E-03 | NELL1 | 7.00E-03 |  |  |
